# A novel in vitro experimental design for biomechanical testing of patellofemoral joint kinetics and kinematics

**DOI:** 10.1051/sicotj/2025043

**Published:** 2025-08-26

**Authors:** A. Mounir Boudali, Jobe Shatrov, Koki Abe, Marcus Zavala, David Parker, William L. Walter, Elizabeth Clarke

**Affiliations:** 1 Murray Maxwell Biomechanics Laboratory, Kolling Institute, Northern Sydney Local Health District 10 Westbourne St St Leonards NSW 2065 Australia; 2 Landmark Orthopaedics Level 2, 500 Pacific Highway St Leonards NSW 2065 Australia; 3 University of Miami, College of Engineering, Department of Mechanical and Aerospace Engineering, Advanced Materials Innovations Laboratory 1600 NW 10th Ave. Miami Fl 33136 USA

**Keywords:** Patellofemoral, Knee, Patellofemoral joint, Knee kinematics, Knee kinetics

## Abstract

*Introduction*: Complications arising from the patellofemoral joint (PFJ) represent the third most common cause for revision in total knee arthroplasty (TKA). Previous in vitro biomechanical studies have altered the native attachments of muscles controlling the PFJ. The purpose of this study was to design an in vitro biomechanical setup that would allow testing of both native and arthroplasty knee joints, specifically the PFJ, without disturbing the native attachments of the quadriceps and hamstrings muscles. *Methods*: After finalising a prototype, a pelvis-to-toe human cadaver specimen was tested. The simVITRO platform was used to simulate movement and control force trajectories. A motion capture system was used to capture the motion of the bones and to measure knee flexion angle and patellar movement with respect to the femur. The forces applied in the PFJ were measured using a custom patella sensor. *Results*: Displacement of the reflective cluster attached to the femur was measured during compression loading at different flexion angles, passive flexion and stairs descent trajectory. The femur showed less than 1 mm and 3 mm displacement with respect to the femur clamp in passive flexion and stairs descent. The most translation of 8.37 mm (<2% average femur length) was observed at 90° flexion which occurred at 483 N simulated compression force. *Conclusion*: This novel design provides a methodology for studying the biomechanics of the PFJ in vitro that preserves the soft tissues influencing the behaviour of the joint. This setup provides a biomechanics model that can be utilised to better understand and study the PFJ in vitro.

## Introduction

Complications arising from the patellofemoral joint (PFJ) represent the third most common cause for revision in total knee arthroplasties (TKAs) in some joint registries. Altered contact pressures and kinematics have been proposed as the aetiology of PFJ dysfunction that leads to symptoms [1–4]. However, the precise effect of surgeon controllable variables that may alter its behaviour remain poorly understood.

Research on the forces applied to the PFJ in-vitro has been limited, primarily due to challenges pertaining the size and complexity of the joint. The simVITRO^®^ Universal Musculoskeletal Simulator (Jupiter 3000, Cleveland Clinic, Cleveland, OH) provides a novel solution, offering the capability of accurately and repetitively simulating a range of motion and loading conditions via precise control of a robotic platform. This setup enables the simulation of the kinetics of the knee joint through a transformation of force and torque data measured by a 6 degree of freedom (DOF) load cell mounted between the specimen and fixed pedestal. The kinematics of the knee and the PFJ are simulated through a data fusion from a robotic arm, and a motion capture system.

The patella kinematics and patellofemoral contact pressures have been studied [5–7]. Previous in vitro biomechanical studies have transected the femur mid-thigh and the tibia mid shin to isolate the knee joint. This disturbs the native attachments of muscles controlling the behaviour of the PFJ, namely the quadriceps muscles and iliotibial band. Whilst investigations have sought to overcome this design problem by a series of pulleys or synthetic materials being attached to act as force actuators, the modelling remains based on many assumptions and disturbs the soft tissue attachments that influence the behaviour of the PFJ.

The purpose of this study was to design an in vitro biomechanical setup that would allow testing of both native and arthroplasty knee joints, specifically the PFJ, without disturbing the native attachments of the quadriceps and hamstrings musculature as well as the iliotibial band.

## Methods

During the design phase, saw-bones were mounted to the testing rig for pilot testing. A testing apparatus was designed to allow for the preservation of the pelvis as well as the foot to satisfy the requirements of a surgical robot that would be used to performed TKA and the biomechanical testing platform. After the final design was complete, a pelvis-to-toe human cadaver specimen was acquired to test and validate the testing apparatus. Ethics approvals were obtained to conduct this study. All research was carried out according to the guidelines for Cadaver Dissection in Education and Research of Clinical Medicine and received ethics approval from the North Sydney Local Health District (NSLHD) ethics committee – approval 2019/ETH08332, general amendment 5 version 1, ID 67876.

### Patellofemoral joint kinetics

The forces applied in the PFJ were measured using a custom patella sensor comprising three uniaxial ultra-thin force sensors (100 um). Each unit was calibrated using a material testing machine Instron 8874 (Instron, Norwood, MA) resulting in a reliable measure of force of up to 600 N. To interface with the trochlear, the sensor was fitted with custom made patellar domes designed to replicate the shape and thickness of commercially available patellar implants. The sensor was connected to a NI Usb-6001 data acquisition board (National Instruments, Austin, TX) and data was streamed and recorded in simVITRO software at 250 Hz.

### Patellofemoral joint kinematics

**Figure 1:**
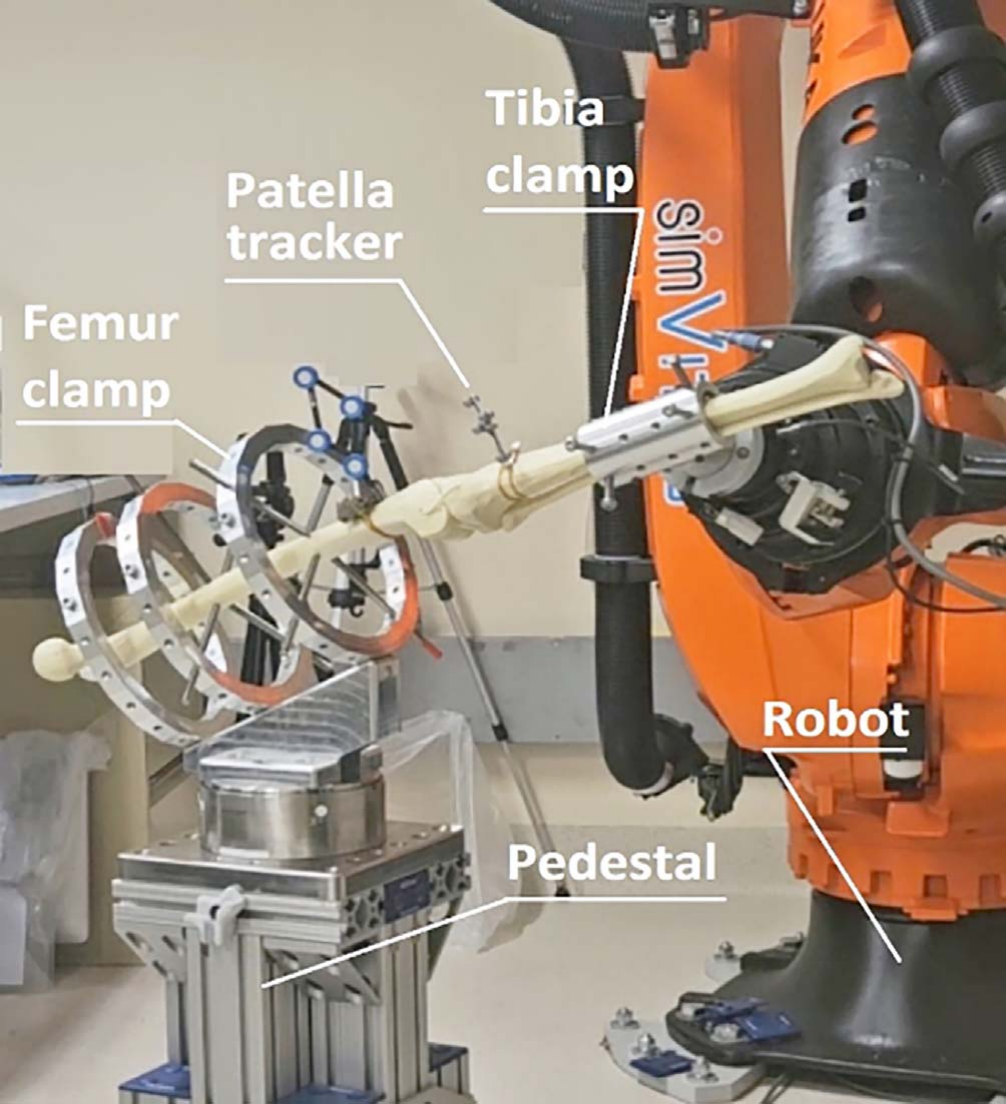
Prototype testing with saw-bone setup. Experimental setup comprising a clamping system fitted to a femur-tibia bone model and a robotic testing machine. The Femur clamp is mounted on a loadcell and securely holds the femur bone in place. The robotic arm holds the Tibia clamp which securely attaches to the tibia bone.

**Figure 2:**
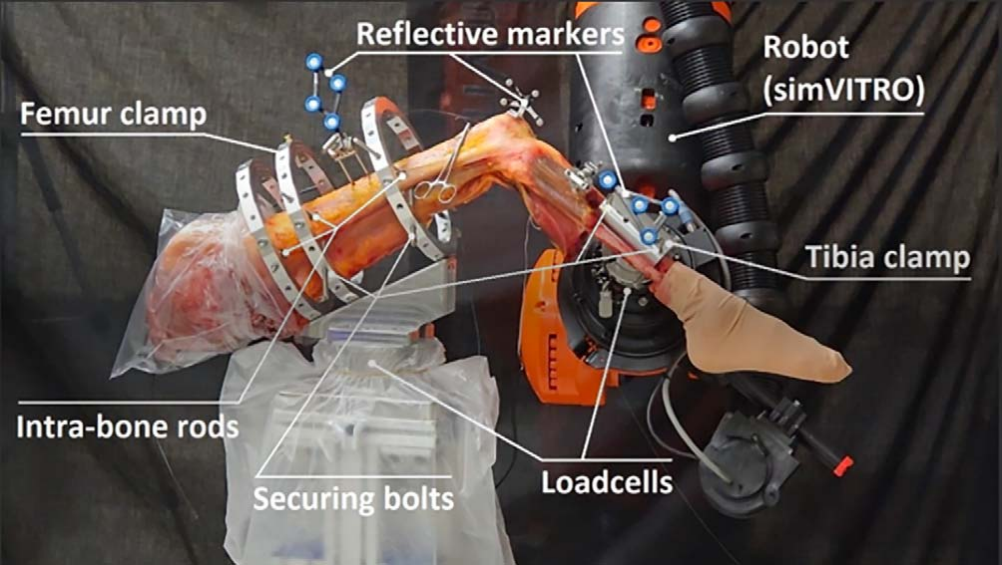
Cadaveric testing setup. A cadaveric limb (pelvis to toe) securely held by the clamping system which attaches to the pedestal and the robotic arm via two loadcells. Three reflective clusters mounted on the femur, the tibia, and the patellar bone.

**Figure 3:**
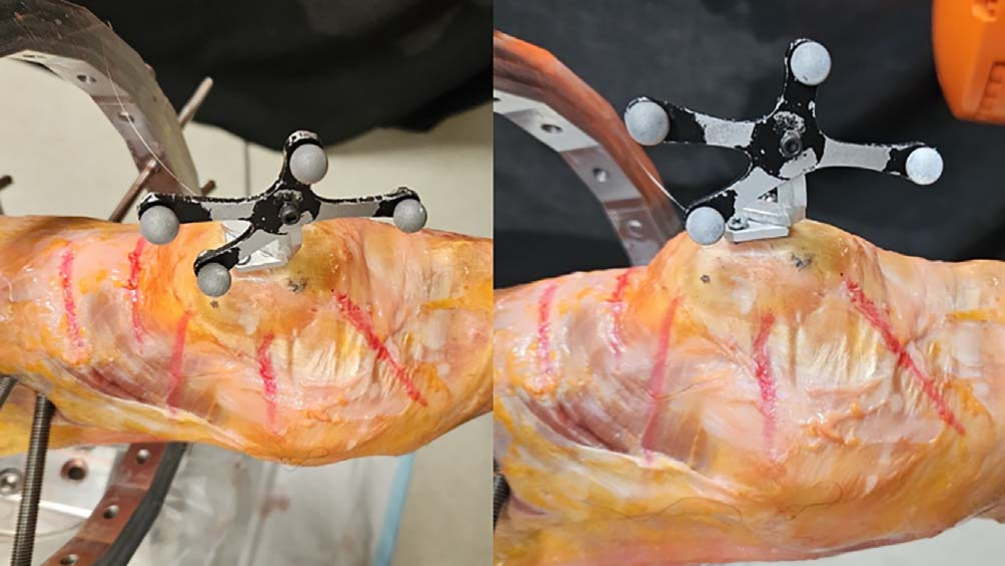
Patella kinematic measurement. A magnetic patella array fitted to a baseplate which is mounted on the patellar bone by way of x2 screws transfixing it to the bone.

A motion capture system (OptiTrack, NaturalPoint Inc., Corvallis, OR) comprised of four wide angle cameras Prime 13 W was used to capture the motion of the bones and to measure knee flexion angle and patellar movement with respect to the femur. These measures were evaluated through the estimation of displacement and rotation of three metallic arrays attached to the Femur, the Tibia, and the Patella bones. These were fixed to the bone by way of 4.0 mm diameter self-tapping metallic pins on the tibia and femur, and 1.5 mm diameter cortical screws on the patella (Figures 1–3). Anatomical landmarks were used to define joint coordinate systems according to Grood and Suntay [8]. The motion capture data were captured at 120 Hz and synchronized with data from the simVITRO platform.

### Movement control

The simVITRO platform was used to simulate movement and control force trajectories. The simVITRO testing platform is a combination of software and hardware designed to perform custom and pre-defined motion trajectories using force control, position control, or a hybrid control. It also collects and processes data from a motion capture system to provide tracking data in the testing joint coordinate system. Trajectories were designed to simulate three motion scenarios. (1) Knee compression at different flexion angles, herein 0°, 30°, 60°, 90°, and 120°, where the compression force target was set to 600 N and the motion halted when torque was approaching the loadcell safety torque limits defined in simVITRO. (2) Passive flexion was designed such that the knee joint flexes from 5° to 100°, holds at maximum flexion for 7 s, then extends back to 5° at a motion rate of 2.2°/s while maintaining 20 N knee compression force and zero force and torque for the reminder degrees of freedom. (3) Stairs descent motion and load profiles were adapted from the Orthoload database, which is the result of studies examining in vivo knee joint kinetics and kinematics using instrumented implants [9, 10]. Forces and torques were scaled to 25% of reported values in Orthoload database to meet robotic system requirements and prevent damaging specimens. Both trajectories were then performed 5 times for each testing condition.

### Clamping apparatus

The testing jig is made of three elements: (1) the femoral clamp is made of a base that forms 30° with transverse plane, allowing for deep knee bend (max 120°) without the tibia, the foot, or the robot colliding with the mounting pedestal (Figure 1). It also comprises a minimum of three rings that can be positioned along the mounting plate. The rings are made of two halves secured with two bolts. Each half ring incorporates a series of M8 holes allowing for the insertion of securing bolts that form contact with the femur bone. (2) The tibia clamp is made of base, and a cylinder split in half which allows the clamping of the tibia without the need to remove the foot nor the fibula. The cylinder has a series of threaded M8 holes through which securing bolts are placed to ensure a rigid fixation of the tibia. The robotic testing arm attaches to the tibia to simulate movement and force trajectories. The fibula was preserved and the ring placed in a manner such that it was not included within the clamp. (3) The patella tracker is made of a plate that mounts on the patella with two small screws and an L shaped plate holding four reflective markers that attaches to the plate using two sets of magnets. The CAD files of the clamping system as well as a bill of material are made available in an online repository.

### Specimen preparation and mounting

The testing setup was designed to control and test cadaveric specimens from hemi-pelvis to toe. The specimen was received as a whole pelvis with both lower limbs attached. After thawing for 48 h, the cadaveric specimen was first separated into two halves by transecting the pelvis through the mid sagittal plane of the sacrum and symphysis pubis to separate each leg. Next, all skin and sub-cutaneous tissue was dissected and removed. No muscles or tendons arising from the pelvis and attaching to the knee were disturbed. For attachment to the tibial holding clamp, all muscle arising from tibia and fibula were transected and removed at a point 17 cm below the knee joint.

To isolate movement at the knee joint, movement at the hip joint was eliminated by way of 2 6.5 mm self-tapping bolts placed in an antegrade fashion through the pelvis into the femoral head whilst it is held in the extended position.

To mount the specimen to the rig, an assistant held the limb in the middle of the rings and a 4.5 mm diameter long drill bit was used to create three pilot holes for placement of the transfemoral rods that connected the femur to jig. The 6 mm diameter threaded transfemoral rods had locking nuts placed to prevent movement of the specimen against the apparatus and increase the stiffness of the testing apparatus. Care was taken to ensure that the rods were placed posterior to the lateral intermuscular septum to prevent any tethering effect to the iliotibial band or quadriceps muscle bellies. To ensure stability of the specimen, additional 8 mm diameter threaded bolts were placed through the rig onto the femoral shaft. These were placed posterior to the lateral intermuscular septum (Figure 2). The patella plate that mounts the patella tracking array was then fixed to the patella by way of two 10 mm long screws (Figure 3). Prior to mounting to the load cell, the specimen was weighed, and the centre of mass (COM) was calculated to apply the required offsets to allow for an accurate simulation of force trajectories and movements by the robotic testing arm in the joint coordinate system.

The specimen with the cadaveric limb (pelvis-to-toe) was then attached to the robotic load cell via a plate holding the ring apparatus (Figures 1 and 2). This was then attached by way of two large bolts that fixed the plate rigidly to the load cell. Finally, the femur, patella and tibia were registered by way of a series of digitised anatomical landmarks to create a joint coordinate system following Grood and Suntay [8].

## Results

We tested and validated the testing apparatus on a pelvis-toe fresh-frozen cadaver specimen of which the femur measured 445 mm in length (greater trochanter to lateral epicondyle).

Displacement of the reflective cluster attached to the femur was measured during compression loading at different flexion angles, passive flexion and stairs descent trajectory adapted from the Orthoload database [4].

**Figure 4:**
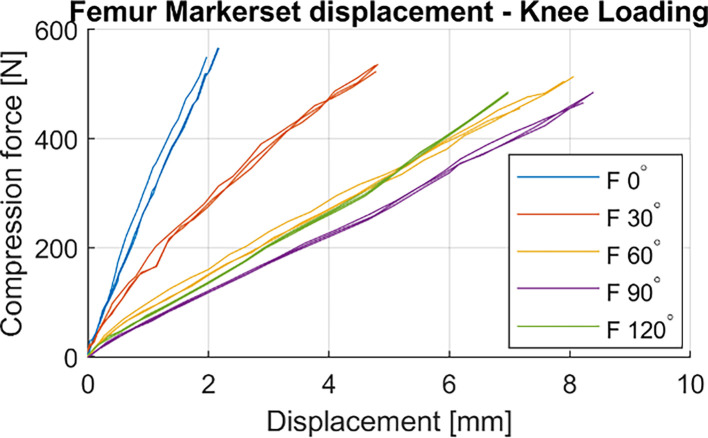
Displacement of the Femur marker set with respect to world frame during knee compression at different knee flexion angles (0, 30, 60, 90, 120) performed on one cadaver specimen. Each line corresponds to one loading cycle.

The femur showed less than 1 mm and 3 mm displacement with respect to the femur clamp in passive flexion and stairs descent, respectively (Table 1). Under load, the most translation of 8.37 mm (<2% average femur length) was observed at 90° flexion which occurred at 483 N simulated compression force through simVITRO (Figure 4).

**Table 1:** Displacement of the femur marker set with respect to world frame during passive flexion and stairs descent.

	Passive flexion	Stairs descent
Knee compression range [N]	−21.29 to 79.07	14.20 to 273.91
Maximum Displacement [mm]	0.94	2.73

Using a calibrated motion capture system, we evaluated the repeatability of the patella tracker removal/placement. We recorded the 6 DOF data of the tracker from 20 cycles of removal and placement of the tracker on the magnetic plate. Results showed variation less than 0.21 mm in *X*, *Y*, and *Z*, and less than 0.14° in Roll and Pitch, and less than 1.37° in Yaw.

## Discussion

Biomechanical testing of the PFJ in vitro is challenging due to the complex geometry of the joint and the nature of the soft tissue attachments. This study described a novel testing setup that provides a method by which the muscle attachments influencing the PFJ arising from the pelvis are left preserved. Minimal translation between the specimen and the clamping apparatus was observed while loading the knee to 600 N. This is a conservative threshold that does not reflect the capabilities of the designed testing apparatus in terms of strength.

**Figure 5:**
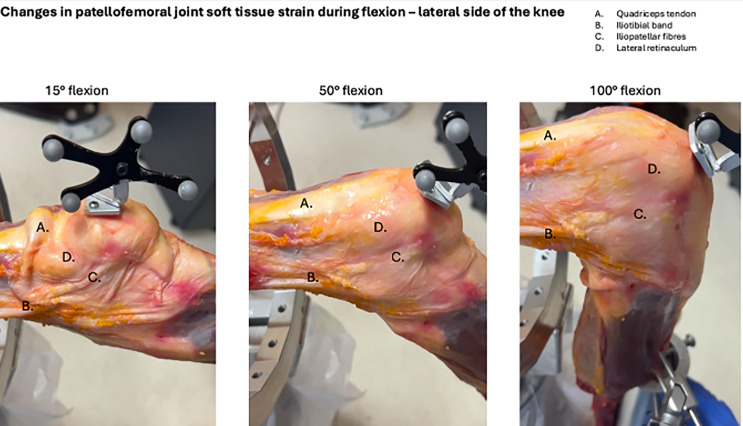
Lateral sided soft tissue strain change during flexion. The soft tissues of the knee undergoing significant changes in tension during flexion as depicted on the lateral side in this image – highlighting the importance of a testing model that preserves their attachments.

The PFJ has a complex soft tissue envelope being made up of static and dynamic stabilisers. The dynamic stabilisers are made up if the quadricep muscle bellies that arise from the femur and pelvis and attach directly to the patella but also interdigitate with the static stabilisers of the PFJ [11, 12]. The static soft tissue stabilisers are the patellar tendon, joint capsule, and ligamentous structures. Medially these include the medial patellofemoral ligament (MPFL), the medial meniscopatellar ligament [13, 14] and the medial retinaculum which interdigitate with the medial collateral ligament and the medial patellar tendon [14]. Laterally, the static soft tissue stabilisers of the PFJ are the patellofemoral ligament, joint capsule, iliotibial band (ITB), and lateral retinaculum. The lateral retinaculum contains a superficial (arciform fibres) and a deep layer (ilio-patella band) that extends from the iliotibial band ITB to the patella [12], a quadriceps expansion and a deep layer that interdigitates with the vastus lateralis, patellofemoral ligament, and patellotibial ligament [15]. The behaviour of the soft tissues throughout flexion changes significantly as demonstrated in Figure 5 where it can be seen the soft tissue structures are generally lax in extension but become progressively tensioned throughout flexion. This demonstrates the importance of preserving the native attachments of the soft tissues that influence the behaviour of the PFJ, which largely arise from the pelvis.

Previous cadaveric biomechanical studies examining the PFJ have opted for models that disturb the native attachments of the quadriceps and hamstring muscles [6, 7, 9, 16–25]. This typically involves removing some of the soft tissue attachments of the patella and sectioning the femur proximal to the knee joint. Whilst this overcomes the complexities of studying a large specimen with several joints, it may alter the behaviour of soft tissue structures directly controlling the PFJ. To overcome this design flaw, a number of studies have attached a pulley system to various muscles with the aim of simulating native muscle vectors. However, such designs may create unnatural behaviour of the of the PFJ and are plagued with issues such as determining the exact vector, the amount of force and reliable attachment of pulley systems to cadaveric tissue. The current model circumvents all such design problems and allows the soft tissue envelope that influences the PFJ to be left untouched, improving the validity of the results.

Patella kinematics in vitro have also previously been studied [10, 26–34]. Despite extensive investigation, data regarding the behaviour of the PFJ in vitro has varied widely. Whilst the reason for such heterogeneity in the literature remains unsolved, it may reflect both the complexity of the joint, but also the varying methodologies that have been utilised in order to overcome the complexities of the PFJ.

This testing setup has limitations. Firstly, to eliminate movement at the hip joint, transarticular bolts were placed with the pelvis in the extended position. This creates an abnormal position of the pelvis for simulated movements such as sit-to-stand where the pelvis would normally go from a flexed to extended position. Nonetheless, biomechanics testing requires the joint being examined to be isolated such that contributions from others joints are eliminated. Furthermore, the force trajectories used in the stairs descent testing condition were scaled down (25%) from physiological values reported in the Orthoload database. This decision was made to preserve the integrity of the specimen and the loadcells.

Studying the PTFJ in TKA remains difficult. Whilst recent advancements in robotic surgery surrounding the PFJ [35, 36] are promising and will no advance knowledge, reliable testing models that allow comparative biomechanical analyses of implant design, positioning and surgical technique are critical to advancing our understanding on this topic. This design we believe will serve as a basis from which many questions can be answered regarding the effect of such variable on the PTFJ in TKA.

## Conclusion

This novel design provides a methodology for studying the biomechanics of the PFJ in vitro that preserves the soft tissues influencing the behaviour of the joint. The design is capable of withstanding more than 600 N knee compression and allowed for the achievement of deep knee bend. This setup provides a biomechanics model that can be utilised to better understand and study the PFJ in vitro.

## Data Availability

All testing data is available on request.

## References

[1] Kemp MA, Metcalfe AJ, Sayers A, Wylde V, Eldridge JD, Blom AW (2018) Does overstuffing of the patellofemoral joint in total knee arthroplasty have a significant effect on postoperative outcomes? Knee 25(5), 874–881. 29933936 10.1016/j.knee.2018.05.007

[2] Barrett D, Brivio A (2023) The third compartment of the knee: an update from diagnosis to treatment. EFORT Open Rev 8(5), 313–318. 37158410 10.1530/EOR-23-0036 PMC10233800

[3] Dye SF (2005) The pathophysiology of patellofemoral pain: a tissue homeostasis perspective. Clin Orthop Relat Res 436, 100–110. 10.1097/01.blo.0000172303.74414.7d 15995427

[4] Scuderi GR, Insall JN, Scott NW (1994) Patellofemoral pain after total knee arthroplasty. J Am Acad Orthop Surg 2(5), 239–246. 10709015 10.5435/00124635-199409000-00001

[5] Huberti HH, Hayes WC (1984) Patellofemoral contact pressures. The influence of q-angle and tendofemoral contact. J Bone Joint Surg Am 66(5), 715–724. 6725318

[6] Lee TQ, Yang BY, Sandusky MD, McMahon PJ (2001) The effects of tibial rotation on the patellofemoral joint: assessment of the changes in in situ strain in the peripatellar retinaculum and the patellofemoral contact pressures and areas. J Rehabil Res Dev 38(5), 463–469. 11732824

[7] Lee TQ, Gerken AP, Glaser FE, Kim WC, Anzel SH (1997) Patellofemoral joint kinematics and contact pressures in total knee arthroplasty. Clin Orthop Relat Res 340, 257–266. 10.1097/00003086-199707000-00033 9224264

[8] Grood ES, Suntay WJ (1983) A joint coordinate system for the clinical description of three-dimensional motions: application to the knee. J Biomech Eng 105(2), 136–144. 6865355 10.1115/1.3138397

[9] Fujikawa K, Seedhom BB, Wright V (1983) Biomechanics of the patello-femoral joint. Part II: A study of the effect of simulated femoro-tibial varus deformity on the congruity of the patello-femoral compartment and movement of the patella. Eng Med 12(1), 13–21. 6682056 10.1243/emed_jour_1983_012_005_02

[10] Heegaard J, Leyvraz PF, Van Kampen A, Rakotomanana L, Rubin PJ, Blankevoort L (1994) Influence of soft structures on patellar three-dimensional tracking. Clin Orthop Relat Res 299, 235–243. 8119024

[11] Fulkerson JP, Gossling HR (1980) Anatomy of the knee joint lateral retinaculum. Clin Orthop Relat Res 153, 183–188. 7449213

[12] Merican AM, Amis AA (2008) Anatomy of the lateral retinaculum of the knee. J Bone Joint Surg Br 90(4), 527–534. 18378934 10.1302/0301-620X.90B4.20085

[13] Reider B, Marshall JL, Koslin B, Ring B, Girgis FG (1981) The anterior aspect of the knee joint. J Bone Joint Surg Am 63(3), 351–356. 7204430

[14] Desio SM, Burks RT, Bachus KN (1998) Soft tissue restraints to lateral patellar translation in the human knee. Am J Sports Med 26(1), 59–65. 9474403 10.1177/03635465980260012701

[15] Powers CM, Landel R, Perry J (1996) Timing and intensity of vastus muscle activity during functional activities in subjects with and without patellofemoral pain. Phys Ther 76(9), 946–955. discussion 956–967. 8790273 10.1093/ptj/76.9.946

[16] Kessler O, Patil S, Colwell CW Jr., D’Lima DD (2008) The effect of femoral component malrotation on patellar biomechanics. J Biomech 41(16), 3332–3339. 19019376 10.1016/j.jbiomech.2008.09.032

[17] Reuben JD, McDonald CL, Woodard PL, Hennington LJ (1991) Effect of patella thickness on patella strain following total knee arthroplasty. J Arthroplasty 6(3), 251–258. 1940931 10.1016/s0883-5403(06)80172-5

[18] Hsu HC, Luo ZP, Rand JA, An KN (1996) Influence of patellar thickness on patellar tracking and patellofemoral contact characteristics after total knee arthroplasty. J Arthroplasty 11(1), 69–80. 8676121 10.1016/s0883-5403(96)80163-x

[19] Matthews LS, Sonstegard DA, Henke JA (1977) Load bearing characteristics of the patello-femoral joint. Acta Orthop Scand 48(5), 511–516. 596148 10.3109/17453677708989740

[20] Fujikawa K, Seedhom BB, Wright V (1983) Biomechanics of the patello-femoral joint. Part I: A study of the contact and the congruity of the patello-femoral compartment and movement of the patella. Eng Med 12(1), 3–11. 6682059 10.1243/emed_jour_1983_012_004_02

[21] Jenny JY, Lefebvre Y, Vernizeau M, Lavaste F, Skalli W (2002) In vitro analysis of the continuous active patellofemoral kinematics of the normal and prosthetic knee. Rev Chir Orthop Reparatrice Appar Mot 88(8), 797–802. 12503021

[22] Ghosh KM, Merican AM, Iranpour F, Deehan DJ, Amis AA (2009) The effect of overstuffing the patellofemoral joint on the extensor retinaculum of the knee. Knee Surg Sports Traumatol Arthrosc 17(10), 1211–1216. 19526222 10.1007/s00167-009-0830-0

[23] Ghosh KM, Merican AM, Iranpour F, Deehan DJ, Amis AA (2010) The effect of femoral component rotation on the extensor retinaculum of the knee. J Orthop Res 28(9), 1136–1141. 20217838 10.1002/jor.21117

[24] Merican AM, Ghosh KM, Baena FR, Deehan DJ, Amis AA (2014) Patellar thickness and lateral retinacular release affects patellofemoral kinematics in total knee arthroplasty. Knee Surg Sports Traumatol Arthrosc 22(3), 526–533. 23271038 10.1007/s00167-012-2312-z

[25] Merican AM, Ghosh KM, Iranpour F, Deehan DJ, Amis AA (2011) The effect of femoral component rotation on the kinematics of the tibiofemoral and patellofemoral joints after total knee arthroplasty. Knee Surg Sports Traumatol Arthrosc 19(9), 1479–1487. 21484388 10.1007/s00167-011-1499-8

[26] Ahmed AM, Duncan NA (2000) Correlation of patellar tracking pattern with trochlear and retropatellar surface topographies. J Biomech Eng 122(6), 652–660. 11192388 10.1115/1.1322036

[27] Ahmed AM, Duncan NA, Tanzer M (1999) In vitro measurement of the tracking pattern of the human patella. J Biomech Eng 121(2), 222–228. 10211457 10.1115/1.2835107

[28] Farahmand F, Tahmasbi MN, Amis AA (1998) Lateral force-displacement behaviour of the human patella and its variation with knee flexion – a biomechanical study in vitro. J Biomech 31(12), 1147–1152. 9882047 10.1016/s0021-9290(98)00125-0

[29] Heegaard J, Leyvraz PF, Curnier A, Rakotomanana L, Huiskes R (1995) The biomechanics of the human patella during passive knee flexion. J Biomech 28(11), 1265–1279. 8522541 10.1016/0021-9290(95)00059-q

[30] Hsu HC, Luo ZP, Rand JA, An KN (1997) Influence of lateral release on patellar tracking and patellofemoral contact characteristics after total knee arthroplasty. J Arthroplasty 12(1), 74–83. 9021506 10.1016/s0883-5403(97)90051-6

[31] van Kampen A, Huiskes R (1990) The three-dimensional tracking pattern of the human patella. J Orthop Res 8(3), 372–382. 2324856 10.1002/jor.1100080309

[32] Bull AM, Katchburian MV, Shih YF, Amis AA (2002) Standardisation of the description of patellofemoral motion and comparison between different techniques. Knee Surg Sports Traumatol Arthrosc 10(3), 184–193. 12012037 10.1007/s00167-001-0276-5

[33] Katchburian MV, Bull AM, Shih YF, Heatley FW, Amis AA (2003) Measurement of patellar tracking: assessment and analysis of the literature. Clin Orthop Relat Res 412, 241–259. 10.1097/01.blo.0000068767.86536.9a 12838076

[34] Nagamine R, Otani T, White SE, McCarthy DS, Whiteside LA (1995) Patellar tracking measurement in the normal knee. J Orthop Res 13(1), 115–122. 7853092 10.1002/jor.1100130117

[35] Shatrov J, Khasian M, Lording T, Monk AP, Parker D, Lustig S (2024) Robotic assessment of patella tracking in total knee arthroplasty. J ISAKOS 9(5), 100287. 38909904 10.1016/j.jisako.2024.06.006

[36] Batailler C, Greiner S, Rekik HL, Olivier F, Servien E, Lustig S (2024) Intraoperative patellar tracking assessment during image-based robotic-assisted total knee arthroplasty: technical note and reliability study. SICOT J 10, 44. 39475330 10.1051/sicotj/2024037 PMC11523864

